# Dataset on non-state actor participation in regional fisheries management organizations

**DOI:** 10.1016/j.dib.2020.106682

**Published:** 2020-12-25

**Authors:** Lisa M. Dellmuth, Matilda T. Petersson, Daniel C. Dunn, André Boustany, Patrick N. Halpin

**Affiliations:** a Department of Economic History and International Relations, Stockholm University, SE-10691 Stockholm, USA; b Stockholm Resilience Centre, Stockholm University, SE-10691 Stockholm, USA; c Marine Geospatial Ecology Lab, Nicholas School of the Environment, Duke University, Durham, NC 27708, USA; d Monterey Bay Aquarium, 886 Cannery Row, Monterey, California 93940, USA

**Keywords:** Environmental non-governmental organizations (ENGOs), Fisheries governance, Institutional change, Non-state actors, Participation, Regional fisheries management organizations (RFMOs)

## Abstract

In this article, we present and describe a new dataset of non-state actor participation in seven regional fisheries management organizations (RFMOs). The dataset contains institutional, economic and ecological variables relevant for non-state actor participation in RFMOs and for RFMO effectiveness. To code non-state actor participation and institutional factors, we quantify information from publicly available RFMO reports as well as data from the Policy IV dataset. We pair these data with existing datasets on ecological and economic factors from the RAM Legacy and the Sea Around Us databases. This article describes the data collection process and the coded variables in detail.

## Specifications Table

**Table:** 

Subject	Social Sciences
Specific subject area	Non-state actor participation in international environmental institutions
	
Type of data	Table
How data were acquired	The data were hand-coded in Excel and transferred to Stata.
Data format	Raw and analysed
	
Parameters for data collection	Institutional, economic and ecological parameters.
Description of data collection	Several researchers were involved in collecting the data. Coding non-state actor participation data in an automatized way was not possible, given the diverse formats of the original files. The data on non-state actor participation and institutional factors were hand-coded from official documents and combined with existing datasets on ecological and economic factors.
Data source location	Primary data sources:
	RFMO official documents
	RAM Legacy database
	Sea Around Us (SAU) database
Data accessibility	The data are available at: https://doi.org/10.7910/DVN/XACJIF
Related research article	Dellmuth, L.M, Petersson, M.T, Dunn D.C., Boustany, A., Halpin P.N. (2020) Empowering NGOs? Long-Term Effects of Ecological and Institutional Change on Regional Fisheries Management Organizations, *Global Environmental Change*, 65, 102,197,
	https://doi.org/10.1016/j.gloenvcha.2020.102197.

## Value of the Data

• The data can be used to enhance knowledge on the patterns, causes, and consequences of non-state actor participation in RFMOs.
• The data can be used in future research on the political dimensions and the effectiveness of RFMOs.
• The data will mainly be of interest to researchers, but can also be used by practitioners interested in illustrating non-state actor participation or other political dimensions of the RFMOs.

## 1 Data Description

We share a replication dataset on non-state actor participation in seven region fisheries management organizations (RFMOs) with the readers. Non-state actors are referred to as profit or non-profit private organizations, such as environmental non-governmental organizations (NGOs), fishing companies, industry associations, private research institutes, and consultancies. In the repository mentioned under ‘Data accessibility’, we include the full dataset in three parts. Observations are coded at the level of non-state actor types, clustered in species that RFMO meetings deal with, RFMOs, and years from 1980 to 2014. Due to the complex hierarchical data structure of the data, we provide the dataset in three parts, each created to facilitate replication of a specific part of the analysis in the original article. We describe the three specific datasets in the following.

The first dataset (engo_by_rfmo.tab) contains a measure of the number of NGOs participating in an RFMO in a given year. It enables a replication of Figure 1 of the related research article about NGO abundance. The second dataset (nsa-all.tab) encompasses information about non-state actor participation in an RFMO in a specific year. For the five non-state actor types introduced in detail below, we include information about the density of their population through a measure of the proportion of specific non-state actor types to all non-state actors. This dataset allows for a replication of Figure 2 of the related research article about the density of non-state actor populations. In the third dataset (nsa_type_by_rfmo.tab), we include the entire dataset at the level of species clustered in RFMOs and years, which is necessary to replicate Figure 3 of the related research article, the regression analysis in Table 2, and the robustness checks of the related research article. This dataset also contains the information about RFMO characteristics and the species covered.

In the remainder of this article, we start by describing the characteristics the seven RFMOs included (Table 1). We then describe the institutional, ecological and economic variables and how they are referred to in the repository (Table 2).

**Table 1:** RFMO characteristics.

Organization name	Member states	Geographical area	Type of mandate	Entered into force	Rules for non-state actor access
Commission for the Conservation of Antarctic Marine Living Resources (CCAMLR)	Argentina, Australia, Belgium, Brazil, Chile, China, European Union, France, Germany, India, Italy, Japan, Namibia, New Zealand, Norway, Poland, Republic of Korea, Russian Federation, South Africa, Spain, Sweden, Ukraine, United Kingdom of Great Britain and Northern Ireland, United States of America, and Uruguay	Southern Ocean	Multiple-species	1982	Non-state organizations may participate as an observer the meetings of the Commission and its subsidiary bodies. Organizations can be denied access if a member of the Commission objects. Organizations can get long-term observer status [5].
Commission for the Conservation of Southern Bluefin Tuna (CCSBT)	Australia, Indonesia, Japan, New Zealand, Republic of Korea, and South Africa.	All waters where the southern bluefin tuna is found	Single-species	1994	Non-state organizations with special competence concerning southern bluefin tuna may participate as an observer the meetings of the of the Commission and its subsidiary bodies. Organizations can be denied access if a member of the Commission objects. Organizations can get long-term observer status allowing them to participate in future meetings [6].
Inter-American Tropical Tuna Commission (IATTC)	Belize, Canada, China, Colombia, Costa Rica, Ecuador, El Salvador, European Union France, Guatemala, Japan, Kiribati, Mexico, Nicaragua, Panama, Peru, Republic of Korea, Taiwan Province of China, United States of America, Vanuatu and Venezuela (Bolivarian Republic of).	Pacific Ocean	Multiple-species (tuna and tuna-like species)	1949	Non-state organizations with recognized experience concerning the Commission and the tuna industry of any of the members, may participate as an observer in the meetings of the Commission and its subsidiary bodies. Organizations can be denied access if one-third of the members of the Commission objects. Organizations can get long-term observer status allowing them to participate in future meetings [7].
International Commission for the Conservation of Atlantic (ICCAT)	Albania, Algeria, Angola, Barbados, Belize, Brazil, Canada, Cabo Verde, China, Côte d'Ivoire, Curaçao, Egypt, El Salvador, Equatorial Guinea, European Union, France, Gabon, Gambia, Ghana, Grenada, Guatemala, Guinea, Guinea-Bissau, Honduras, Iceland, Japan, Liberia, Libya, Mauritania, Mexico, Morocco, Namibia, Nicaragua, Nigeria, Norway, Panama, Philippines, Republic of Korea, Russian Federation, Saint Vincent and the Grenadines, Sao Tome and Principe, Senegal, Sierra Leone, South Africa, Syrian Arab Republic, Trinidad and Tobago, Tunisia, Turkey, United Kingdom of Great Britain and Northern Ireland, United States of America, Uruguay, Vanuatu and Venezuela (Bolivarian Republic of).	Atlantic Ocean	Multiple-species (tuna and tuna-like species)	1969	Non-state organizations with a demonstrated interest in the species managed by the Commission may participate as an observer in the meetings of the Commission and its subsidiary bodies. Organizations can be denied access if one-third of the members of the Commission objects [8].
Indian Ocean Tuna Commission (IOTC)	Australia, China, Comoros, Eritrea, European Union, France (overseas territories), Guinea, India, Indonesia, Iran (Islamic Republic of), Japan, Kenya, Madagascar, Malaysia, Maldives, Mauritius, Mozambique, Oman, Pakistan, Philippines, Republic of Korea, Seychelles, Sierra Leone, Somalia, South Africa, Sri Lanka, Sudan, Thailand, United Kingdom of Great Britain and Northern Ireland (overseas territories), United Republic of Tanzania, and Yemen.	Indian Ocean	Multiple-species (tuna and tuna-like species)	1996	Non-state organizations with special competence in the field of activity of the Commission may participate as an observer in the meetings of the Commission and subsidiary bodies. Organizations can be denied access if one of the Members of the Commission objects whereby the matter will be subject to decision of the Commission out of session by written procedure [9].
Northwest Atlantic Fisheries Organization (NAFO)	Canada, Cuba, Denmark (in respect of the Faroe Islands and Greenland), European Union, France (in respect of Saint Pierre and Miquelon), Iceland, Japan, Norway, Republic of Korea, Russian Federation, Ukraine and United States of America.	Atlantic Ocean	Multiple-species	1979	Non-state organizations that supports the general objectives of NAFO and with a demonstrated interest in the species under the purview of NAFO, may participate as an observer in the meetings of the Commission and subsidiary bodies. Organizations can be denied access if one or more of the Contracting Parties objects and gives in writing its reasons, whereby the matter will be put to a vote by written procedure. Organizations can get long-term observer status allowing them to participate in future meetings [10]
Western and Central Pacific Fisheries Commission (WCPFC)	Australia, Canada, China, Cook Islands, European Union, Fiji, France, Indonesia, Japan, Kiribati, Marshall Islands, Micronesia (Federated States of), Nauru, New Zealand, Niue, Palau, Papua New Guinea, Philippines, Republic of Korea, Samoa, Solomon Islands, Taiwan Province of China, Tonga, Tuvalu, United States of America, and Vanuatu.*	Pacific Ocean	Multiple-species (tuna and tuna-like species)	2004	Non-state organizations concerned with matters relevant to the convention, with a demonstrated interest in matters under consideration by the Commission may participate as observers in the Commission its subsidiary bodies. Organizations can be denied access if a majority of the members of the Commission objects to the request. Organizations can get long-term observer status allowing them to participate in future meetings [11].

sNotes: Authors’ own compilation using Convention texts. Source: Rules of Procedures and websites of the RFMOs. * WCFPC also has seven participating territories: American Samoa, French Polynesia, Guam, New Caledonia, Northern Mariana Islands, Tokelau and Wallis and Futuna Islands.

**Table 2:** Variable descriptions.

Concept	Variable in repository
Ecological factors	
Fishing pressure	ffmsy
Fishing pressure weighted by landed value (log)	ffmsylan
Fishing pressure growth	ffmsygr
Biomass status	bbmsy
Institutional factors	
RFMO budget (log)	lbudget_cpi1000
NGO abundance	engo
NGO density	engopropp
Consultancy density	consultancypropp
Industry density	industrypropp
Expert density	researchpropp
Member state diversity	dem_sd
Control variable	
Landed value (log)	llanded_valton

Notes*:* The repository contains a codebook that describes all variables. Variable and value labels can be seen directly in the datasets if the Stata binary files are opened with Stata, R or SPSS. The replication code is written both in R scripts and in Stata do-files.

## 2 Materials and Methods: selection of RFMOs

The dataset covers all RFMOs for which we were able to find availability of information about non-state actor participation in meetings as well as stock assessment data for a sufficiently long time period. This was the case for seven RFMOs (Table 1).

For example, there are no publicly available stock assessment data for the following RFMOs: Convention on the Conservation and Management of Pollock Resources in the Central Bering Sea (CCBSP), General Fisheries Commission for the Mediterranean (GFCM), North Atlantic Salmon Conservation Organization (NASCO), North Pacific Anadromous Fish Commission (NPAFC), South East Atlantic Fisheries Organisation (SEAFO). At the time of coding, these RFMOs had yet to perform stock assessments, and instead used other measures, such as catch per unit effort (CPUE) as fisheries indicators for management. More recently created RFMOs, i.e. Southern Indian Ocean Fisheries Agreement (SIOFA), South Pacific Regional Fisheries Management Organisation (SPRFMO), and North Pacific Fisheries Commission (NPFC) are not included in the dataset, since the former two RFMOs held their first Commission meeting in 2013 and the latter in 2015. The North-East Atlantic Fisheries Commission (NEAFC) and International Pacific Halibut Commission (IPHC) were also excluded, although stock assessment data are available, since neither of these RFMOs record the organizational affiliations of non-state actors attending their meetings.

The dataset contains observations at the level of fish stocks managed by an RFMO in a given year. Not all species managed by the RFMOs are included in the dataset, due to limitations in stock assessment data for some species. More specifically, the dataset covers all species in the Commission for the Conservation of Southern Bluefin Tuna (CCSBT), which is a single-species RFMO. Multiple-species RFMOs apply to all marine living resources or, all tuna and tuna-like species (for the tuna RFMOs) within the specified Convention area, which makes it difficult to assess the total number of species that should be managed by these RFMOs and to assess how many of these are covered by our dataset. Using information about which species these RFMOs cover as stated either in Convention texts or on web sites, we were able to estimate that the dataset covers 11 out of 16 species in IOTC,^1^ 5 out of 11 species in NAFO,^2^ 1 out of 4 in CCAMLR,^3^ and 9 out of 30 in ICCAT.^4^ For IATTC and WCPFC, such information was not available, and we are thus unable to estimate how many species these RFMOs manage.

The dataset presented in this article covers institutional, economic and ecological variables. We coded three institutional factors pertaining to non-state actor participation, RFMO budgets and membership composition, using publicly available information from RFMO annual reports and meeting documents, as well as information from the Polity IV database [12]. We pair these data with information from existing datasets. We use the RAM legacy database [13] to code for ecological factors (i.e., related to fishing pressure and biomass) and the SAU database [14] to code for economic factors (i.e., landed value). In the following, we describe the coding process for each of these variables.

## 3 Institutional and Economic Variables

### 3.1 Non-State actor participation

Non-state actor participation was coded using information from the lists of participants from annual meeting reports of the commissions (decision-making bodies) and the scientific committees (advisory bodies) of the seven RFMOs (Table 1). To ensure that all non-state actor participants are included, we coded both those attending RFMO meetings as accredited observers and as invited experts or advisers of member states and cooperating non-member state delegations [15]. The meeting reports containing the lists of participants are publicly available on the web pages of the studied RFMOs. Where meeting reports or lists of participants were not available, we received this information from the RFMO secretariat upon request. In case the RFMO bodies held several meetings in the same year, which was for example the case in IATTC in some years, we coded the average values of participants over the course of that year.

Non-state actor participation variables were hand-coded. We tried to extract the data using a Python script, but due to the diverse formatting of the original files, this turned out less efficient than hand coding. We attempted a number of other automated methods to identify proper names and organizations from participant lists, including natural language processing. While there were some promising results, the original reports did not present the data in enough of a consistent structure to extract the information with more accuracy than through manual effort. Because PDF documents contain only visual markup for text, and no logical formatting, even tabular data was difficult to parse automatically. Simply breaking up the text by honorific (i.e. Mr, Ms, Dr) was the most effective way to isolate records, but further tagging of proper names and associations was impossible without significantly training the natural language processing tools.

We coded five types of non-state actors: environmental non-governmental organizations (ENGOs), representatives from the fishing industry (i.e., fishing companies and industry associations), private research institutes, and consultancies. For detailed illustrations of these actor categories, we refer the reader to our article for this dataset as well as our previous work [15].

For each non-state actor category, we created two variables: abundance and density. Abundance refers to the total number of actors in a category participating in RFMO meetings. Density is defined as the number of actors in a specific category relative to the number of other participating non-state organizations in an RFMO in a given year.

### 3.2 RFMO resources

RFMOs’ resource endowment is captured by coding annual budgets from RFMO annual reports for the previous year. For example, the 2004 proposed budget was identified in the 2003 annual report. Budget is measured in 2010 constant 1000 dollars to uncover ‘real’ budget development. For this purpose, the budget measure was converted into USD and then divided by the Consumer Price Index, derived from the World Bank database [16].

### 3.3 Member state composition

A variable measuring the diversity of RFMO member states’ political systems was created. Specifically, we measure how far member states differ in terms of levels of democracy, as states differing in terms of democracy levels might have different interests and priorities. For this purpose, we code the standard deviation of countries’ degree of democracy in the Polity IV dataset [12]. This variable captures three essential, interdependent elements of democracy. One is the presence of institutions and procedures through which citizens can express effective preferences about alternative policies and leaders. Second is the existence of institutionalized constraints faced by executives. Third is the guarantee of civil liberties to all citizens in their daily lives and in acts of political participation. Other aspects of plural democracy, such as the rule of law, systems of checks and balances, freedom of the press, are means to, or specific manifestations of these general principles.

### 3.4 Landed value

We extracted data on landed values for all of the species included in the SAU database [14]. The SAU database combines official reported catches from the Food and Agriculture Organization of the United Nations FishStat database [17] and reconstructed estimates of unreported catches (including discards) for individual countries’ Exclusive Economic Zones. The landed value data per species is calculated using official reported values of fish caught and ex-vessel prices, i.e. the amount paid for the fish at the dock as the fish enters the seafood supply chain, as first described by Sumaila et al. [18] and updated by Swartz et al. [19] and Tai et al. [20]. The landed value in the SAU database has been converted to 2010 USD equivalents to allow for comparisons over time. After extracting landed value data on the relevant species between 1980 and 2014, we created a mean landed value for each species in a given year.

## 4 Ecological Factors

We extracted fishing pressure (F/F_MSY_) and biomass status (B/B_MSY_) measures from RFMO stock assessments, available through the RAM legacy database [13]. Our dataset thus captures trends in current stock status relative to agreed targets within the RFMO. The Northwest Atlantic Fisheries Organization reported SSB_MSY_ for two of its managed stocks (American plaice NAFO-5YZ and Atlantic cod NAFO 2J3KL). SSB_MSY_ is analogous to B_MSY_ but only considers spawning stock biomass (the biomass of adult fish) as opposed to the biomass of the entire population. Moreover, information was not available for all species, as the RFMOs do not perform proper stock assessments for all species under their mandate.

Taken together, this dataset is useful for practitioners and researchers interested in analysing the participation of non-state actors in RFMOs, and examining institutional, economic and ecological conditions for RFMO effectiveness.

## Ethics Statement

We retrieved the data from official non-anonymized records. No ethical concerns.

## Credit Author Statement

Lisa M. Dellmuth: Conceptualization, Methodology, Investigation, Writing - Original Draft, Writing - Review & Editing, Visualization. Matilda T. Petersson: Conceptualization, Methodology, Investigation, Writing - Original Draft, Writing - Review & Editing. Daniel Dunn: Methodology, Investigation, Writing - Review & Editing. André Boustany: Methodology, Investigation, Writing - Review & Editing. Patrick Halpin: Methodology, Writing - Review & Editing.

## Declaration of Competing Interest

The authors declare that they have no known competing financial interests or personal relationships which have or could be perceived to have influenced the work reported in this article.

## Footnotes

1 IOTC specifies sixteen species covered by its Convention [1].

2 NAFO states that it manages eleven species on their website [2].

3 CCAMLR specifies that four species are targeted by fisheries within the Convention area on their website [3].

4 ICCAT states that about thirty species are of direct concern to the commission on their website [4].
